# What every intensivist must know about antimicrobial stewardship: its pitfalls and its challenges

**DOI:** 10.5935/0103-507X.20200037

**Published:** 2020

**Authors:** José-Artur Paiva, Paulo Mergulhão, Jorge Ibrain Figueira Salluh

**Affiliations:** 1 Departamento de Medicina Intensiva, Centro Hospitalar Universitário São João - Porto, Portugal.; 2 Departamento de Medicina, Faculdade de Medicina, Universidade do Porto - Porto, Portugal.; 3 Grupo de Infecção e Sepsis - Porto, Portugal.; 4 Instituto D’Or de Pesquisa e Educação - Rio de Janeiro (RJ), Brasil.; 5 Programa de Pós-Graduação em Clínica Médica, Universidade Federal do Rio de Janeiro - Rio de Janeiro (RJ), Brasil.

The increased rates of multiresistant pathogens are a global threat to the healthcare system. As the delivery of new antimicrobials is far from addressing all resistance challenges, new alternatives to improve the current use of antimicrobials have been increasingly studied and adopted. One of the most relevant is undoubtedly antimicrobial stewardship (AMS). Antimicrobial stewardship is increasingly used to improve adherence to guidelines and clinical outcomes, to decrease antimicrobial resistance and other collateral damage and to control costs.

A recent Cochrane review investigated the effectiveness and safety of AMS and found high-certainty evidence that interventions lead to more hospital patients receiving the appropriate treatment according to antibiotic policies and moderate-certainty evidence that interventions reduce the length of hospital stay without affecting safety.^(1)^

Several pitfalls and limitations exist that may compromise the proper conception, implementation and development of AMS for intensive care units (ICU). The purpose of this paper is to briefly address some of the main pitfalls.

## Antimicrobial stewardship should not be performed addressing cost containment as the main purpose

The absence of a universal definition for AMS combined with the lack of international guidance and standards are among the many barriers to the implementation of these programs globally. The purpose of AMS is to promote the optimal/prudent/responsible use of antibiotics to optimize patient outcomes while at the same time minimizing the probability of adverse effects, including toxicity and the selection of potential pathogenic organisms (such as fungi or *Clostridium difficile*), and the emergence and spread of antibiotic resistance, ideally minimizing costs.^(2-4)^

Since the first time it was mentioned in a paper by John McGowan and Dale Gerding, two internists at the Emory University School of Medicine,^(5)^ AMS was seen as a “...large-scale, well-controlled trials of antimicrobial use regulation employing sophisticated epidemiologic methods, molecular biological organism typing, and precise resistance mechanism analysis [...] to determine the best methods to prevent and control antimicrobial resistance and ensure our optimal antimicrobial use stewardship”. These authors also stated that “...the long-term effects of antimicrobial selection, dosage, and duration of treatment on resistance development should be a part of every antimicrobial treatment decision”. Recently, the ESCMID Study Group for Antimicrobial Stewardship^(2)^ emphasized this ecological purpose by defining AMS as a strategy that includes “a coherent set of actions which promote using antimicrobials in ways that ensure sustainable access to effective therapy for all who need them”.

In fact, preserving the effectiveness of the antibiotic is a fundamental and difficult challenge in the context of a global increase and geographic convergence in antibiotic consumption.^(6)^ Although antibiotic consumption rates in most low-middle-income countries remain lower than in high-income countries, despite higher bacterial disease burden, consumption in low-middle-income countries is rapidly converging to rates similar to those in high-income countries.^(7)^ Globally, antibiotic consumption, expressed in defined daily doses (DDD), increased 65%, and the antibiotic consumption rate increased 39% between 2000 and 2015.^(6)^ It is expected that, assuming no policy changes, antibiotic consumption by 2030 will be 200% higher than in 2015.^(6)^

Therefore, cost containment must not be seen as the main purpose of AMS. Preserving antibiotic effectiveness is the seminal and main purpose of minimizing the induction and selection of antimicrobial resistance.

## Antimicrobial stewardship should not be performed based solely or mainly on restrictive interventions

There are three main kinds of AMS interventions: restrictive, enabling and structural.^(8-10)^ Most structural interventions have an enabling nature, for instance, the implementation of better and faster diagnostic methods, the use of antimicrobial resistance and antibiotic consumption surveillance systems or the use of computerized antibiotic decision support systems. With restrictive interventions, such as formulary restrictions, preapproval by senior AMS doctors and automatic stop orders, one tries to reduce the number of opportunities for inadequate prescription while enabling interventions, such as education of prescribers, implementation of treatment guidelines, promotion of de-escalation, *pharmacokinetic*-pharmacodynamic guided dose and dose interval and prospective audit and feedback to providers, aimed at increasing the number of opportunities and decreasing barriers for optimal prescription.

Both enablement and restriction interventions were independently associated with a 15% increase in compliance with desired practice, a 1.95-day decrease in the duration of antibiotic treatment, and a 1.12-day decrease in inpatient length of stay, without compromising patient safety.^(1)^ Restrictive interventions may be risky in some settings, such as the ICU, where prompt access to antibiotics is paramount, and in real life, potential delays in initiating antibiotic treatment were observed with some restricting interventions. They may also be detrimental to the communication between the clinical and stewardship teams. Enabling interventions consistently increases the effect of interventions, enhancing their sustainability, including those with a restrictive component (high‐certainty evidence), and they are associated with better acceptance.^(1)^

Antimicrobial stewardship should be a multifaceted approach in which enabling interventions are fundamental, as they are associated with better clinician acceptance and increase the effect and sustainability of other interventions. Unlike restrictive interventions, enabling does not endanger or delay access to appropriate antibiotic therapy or disrupt communication between clinical and stewardship teams.

## Antimicrobial stewardship should not be performed by omitting behavioral interventions

Education is an important and necessary tool in AMS. In a recent study including 7,328 responses from 179 medical schools in 29 countries,^(11)^ most final-year European medical students feel that they need more education on antibiotic use for their future practice as junior doctors. The proportion of students wanting more education on prudent antibiotic use or general antibiotic use ranged from 20.3% (Sweden) to 94.3% (Slovakia), with a mean of 66.1%, and was strongly inversely correlated with preparedness scores. Higher prevalence rates of antibiotic-nonsusceptible bacteria were associated with lower preparedness scores and higher self-reported needs for further education (p < 0.01).

The recent Cochrane review of 221 studies shows that a majority of programs used educational interventions and that behavioral interventions are seldom used.^(1)^ Behavioral and social science research is underutilized in the development of antimicrobial prescribing interventions. Although feedback further increased the intervention effect, and although enabling interventions that included feedback were more effective than those that did not (moderate‐certainty evidence), feedback was used in only a minority of enabling interventions.

In addition, one should remember that the diagnosis of infection in critically ill ICU patients is frequently riddled with difficulties and uncertainty. This can lead to the overdiagnosis of sepsis and unnecessary antibiotic use, which can be associated with worse outcomes.^(12,13)^ In this setting, behavioral interventions aimed at changing the way clinicians approach suspected infection in the ICU are more likely to have persistent beneficial effects than simpler restrictive measures. For example, the “watchful waiting” strategy initially proposed by Yu et al.^(14)^ and recently reviewed by Denny^(15)^ may be an attractive option in the ICU. Close monitoring of multiple physiological parameters can facilitate the withholding of empirical antibiotics in patients considered to be at low risk of adverse effects if the attending physicians view the reduced exposure to antibiotics as a desirable outcome.

Therefore, both educational and behavioral interventions should be included in AMS programs. Qualitative evidence highlights the influence of social norms, attitudes, and beliefs on antimicrobial prescribing behavior. Behavioral interventions, such as decision aids, desired action as the default option, the use of nudges, and pathways designed based on habits and patterns, require the development of systems that address human factors. The lack of this behavioral approach may be a factor contributing to the challenges that beset interventions aiming to influence prescribing behavior and optimize the prescription of antimicrobials.^(16,17)^ In this scenario, information technology may play an important role, and the use of electronic dashboards as well as nudges should be encouraged.^(18,19)^

The use of near real-time information is feasible through the interoperability of different informatics systems and antibiotic/pathogen dashboards. This approach has a “feedback effect” and may substantially improve knowledge of resistance patterns and better inform the decision to use empirical antibiotics in the ICU. With the increased use of electronic medical records and streaming analytics, dashboards can be made available for ICU physicians with near real-time updates.^(20,21)^ An ideal dashboard should provide information on the actual resistance patterns as well as the dynamics of these patterns, the use of antimicrobials, adherence to processes of care (including local antibiotic guidelines) and healthcare-associated infections. This technology-based element should be coupled with behavioral interventions.

Despite evidence demonstrating the impact of behavioral determinants and social norms on prescribing, they are often not given adequate consideration in the design and evaluation of interventions. The incorporation and application of behavioral sciences supported by appropriate multidisciplinary collaboration is recommended. Usual behavior must be investigated as a first step to changing it, with recognition of the personal, social and environmental factors affecting behavior. In fact, to influence the antimicrobial prescribing of individual healthcare professionals and eventually change culture, interventions need to address prescribing etiquette and use clinical leadership within existing clinical groups to influence practice.^(21)^

## Antimicrobial stewardship should not be designed as an external intervention and omit the definition of locally customized goals

As expected, different AMS interventions have different levels of evidence and different likelihoods of efficacy (Table 1). However, interventions for a specific AMS program should be chosen mainly considering the analysis of the local system. Customization is essential for effectiveness. The weight of the several components of the program should be chosen on the basis of the fundamental specific goals, taking into account local MDR rates, the context/culture of the services that shape the behaviors of the healthcare workers, the main determinants of antibiotic prescription and the resources available for AMS. It is essential to identify barriers and facilitators that impact the successful implementation of recommendations to design a structured plan to address and overcome these barriers.^(22)^ Gabbay et al.^(23)^ showed that clinicians rarely access and use explicit evidence from research or other sources directly but rather mostly rely on “mindlines” - collectively reinforced, internalized and tacit guidelines. They showed that mindlines were mainly informed by their own and their colleagues’ experiences, their interactions with each other and their interactions with opinion leaders, patients and pharmaceutical representatives. They tend to be iteratively negotiated with a variety of key actors, often through a range of informal interactions in fluid “communities of practice”. Pakyz et al., in a qualitative study using semistructured telephone interviews with 15 AMS member pharmacists and six physicians representing 21 unique academic medical centers,^(24)^ demonstrated that successful implementation of AMS strategies was found to be related to communication style, types of relationships formed between the AMS and non-AMS personnel, and conflict management. Success was also influenced by the availability of human and health information technology and the ability to generate and analyze AMS-specific data.

**Table 1:** Likelihood of the efficacy of different antimicrobial stewardship interventions in the intensive care unit

1. Highest Level of Evidence
Prospective audit and feedback of antimicrobial prescriptions
Therapeutic drug monitoring for vancomycin, aminoglycosides, azoles and, if possible, beta lactams
Formulary restrictions
Use of knowledge on local epidemiology and local antibiograms
Partnership with infection and antimicrobial resistance control units or infectious disease experts/services
Systematic and organized communication with microbiology laboratories and microbiologists and with clinical pharmacology units
Swift communication with pharmacy
2. Of likely benefit
Educational programs for all providers
Guideline implementation
De-escalation strategies
Use of rapid diagnostic tests, in accordance with local consensus guidelines

Source: adapted from Kollef MH, Bassetti M, Francois B, Burnham J, Dimopoulos G, Garnacho-Montero J, et al. The intensive care medicine research agenda on multidrug-resistant bacteria, antibiotics, and stewardship. Intensive Care Medicine. 2017;43(9):1187-97.^(10)^

Moreover, as it is largely supported in the literature and by national organizations including Public Health England, British Society for Antimicrobial Chemotherapy and major international governments and organizations, AMS requires cross-specialty collaboration and clinical service engagement. The promotion of leadership from within cohorts internally drives behavior change towards antimicrobial use. The AMS topic must be part of the agenda of each clinical service that must engage in the definition of goals and targets and in the intervention design, implementation and monitoring. The hospital AMS unit must understand that leadership is not wielding authority but rather empowering people and that, as in any quality improvement program, the central AMS unit must move from holding power to sharing power and finally to handing power to peripherally intervening units.

Therefore, there is no gold standard stewardship model. A national guideline is helpful, but effective implementation must be customized to each of the services intervened. Tailored approaches are required, defining organizational and cultural determinants, to ensure that antimicrobial stewardship is effectively implemented everywhere.

## Antimicrobial stewardship should not be implemented and performed outside the frame of a quality improvement initiative

In each hospital, AMS should be viewed as a series of customized quality improvement initiatives linked by a common global goal. Quality improvement initiatives should follow a prespecified improvement cycle that includes extensive planning but also measure the results of the implemented actions. As with any quality improvement initiative, AMS needs the following: a customized target and time-to-target definition, including documentation of the specific prescribing behavior that was targeted by the intervention; a change package; a feedback structure that allows the provision of a summary of clinical performance of healthcare over a specified period of time; and action planning, with the identification of quality gaps, room for improvement, rewards or motivations for meeting a target and the use of action plans if the target was not met. Further improvement may be achieved with additional behavior change intervention functions in a series of levered Plan-Do-Study-Act cycles.

In 2016, a consensus expert panel on metrics assessing the impact of stewardship interventions on a patient level in an acute-care setting was assembled and defined a set of process and outcome indicators ready for immediate use and tracking.^(25)^ However, metrics and indicators should be defined locally and should differ by setting, as goals may and should also be diverse.

Regardless, the implementation and use of a database that provides continuous feedback of metrics and indicators to providers is fundamental but often a real-life problem. In general, surveillance metrics are derived from separate electronic data sources: antibiotic prescription data (pharmacy-based), microbiology results (laboratory-based), diagnostic codes (administration-based) or a combination thereof. Surveillance is not the primary purpose of these sources of information, and using them often results in poor matching of antibiotic prescription data with the corresponding clinical and microbiological information. Their ability to represent the complex nature of the antibiotic treatment decision-making process, and thus their practical usefulness to provide insight into antibiotic prescription, is limited. With the linking of these sources, ideally inside the electronic health record, a deeper understanding of the different factors driving antimicrobial use can be obtained. Interfacing electronic health records, laboratory systems and specialized or general (but customized) analytics tools has become a more common and widely implemented strategy.

Recently, De Bus et al. described the system used in the Ghent University Hospital ICU.^(26)^ Antibiotic prescription data were prospectively merged with diagnostic and microbiology data by ICU physicians through dedicated software during a 4-year period. Definite focus of infection and probability of infection (high/moderate/low) were reassessed by dedicated ICU physicians at patient discharge. By prospectively combining antibiotic, microbiology and clinical data, a longitudinal, multifaceted dataset on antibiotic use/infection diagnosis was constructed. Namely, 80% of antibacterial days of therapy were linked with infection, the predominant foci being the respiratory tract (49%), that a microbial cause was identified in 56% of the cases and that moderate/low probability infections accounted for 42% of antibacterial days of therapy for respiratory tract infections. This allowed the identification of antibiotic prescription patterns that require future antibiotic stewardship attention.

In our opinion, AMS should always be viewed as a quality improvement initiative, requiring an evidence-based, ideally bundled, change package; a clear definition of locally adapted goals, indicators and targets; a dynamic measurement and data collection system with feedback to prescribers on a continuous basis; a strategy for building capacity (available expertise, education, practical training, role-modeling); a plan to identify and approach areas for improvement and address quality gaps using educational and behavioral interventions; and ICU leadership commitment and staff engagement, with accountability and responsibilities.

## Antimicrobial stewardship should not be performed without the engagement of consumers

There is good evidence that public campaigns promoting responsible antibiotic use may be associated with reductions in overall antibiotic use.^(27)^ The individual impact of various public campaigns in Europe between 1997 and 2007 has been estimated to be equivalent to a 6.5% to 28.3% drop in the mean level of overall antibiotic use.^(28)^ Factors leading to successful awareness campaigns include the following: targeting a wide audience, such as families, patients, healthcare workers, community pharmacists and policymakers; using mass and social media; repeating simple key messages; and maintaining the diffusion of these messages even after the end of the campaign, thus globally improving health literacy. In fact, sustaining the impact obtained is the main difficulty,^(29)^ but this objective may be achieved by incorporating this concept into the clinician-patient relationship, namely, providing patients and families with information on treatment options, including evidence of effectiveness and likely benefits and risks of harm to support patient engagement and shared decision making.^(30)^ Consumer representation on antimicrobial stewardship committees may also enable effective communication.

## CONCLUSION

Antimicrobial stewardship should be performed addressing preservation of the effectiveness of the antibiotic as the main purpose; as a multifaceted intervention, using structural, restrictive and enabling strategies; using both educational and behavioral interventions and information technology-based dashboards; as an intervention driven by the service/intensive care unit intervention, building capacity and progressively handling leadership from the antimicrobial stewardship central unit to the local team; by defining customized, local goals and promoting constant feedback and closing the gap; as a quality improvement initiative; and involving consumers (patients and families) and society as a whole.

## References

[1] Davey P, Marwick CA, Scott CL, Charani E, McNeil K, Brown E (2017). Interventions to improve antibiotic prescribing practices for hospital inpatients. Cochrane Database Syst Rev.

[2] Dyar OJ, Huttner B, Schouten J, Pulcini C, ESGAP (ESCMID Study Group for Antimicrobial stewardshiP) (2017). What is antimicrobial stewardship. Clin Microbiol Infect.

[3] Hulscher ME, Prins JM (2017). Antibiotic stewardship: does it work in hospital practice? A review of the evidence base. Clin Microbiol Infect.

[4] Cox JA, Vlieghe E, Mendelson M, Wertheim H, Ndegwa L, Villegas MV (2017). Antibiotic stewardship in low- and middle-income countries: the same but different?. Clin Microbiol Infect.

[5] McGowan JE Jr, Gerding DN (1996). Does antibiotic restriction prevent resistance. New Horiz.

[6] Klein EY, Van Boeckel TP, Martinez EM, Pant S, Gandra S, Levin SA (2018). Global increase and geographic convergence in antibiotic consumption between 2000 and 2015. Proc Natl Acad Sci U S A.

[7] Van Boeckel TP, Gandra S, Ashok A, Caudron Q, Grenfell BT, Levin SA (2014). Global antibiotic consumption 2000 to 2010: an analysis of national pharmaceutical sales data. Lancet Infect Dis.

[8] Pulcini C, Binda F, Lamkang AS, Trett A, Charani E, Goff DA (2019). Developing core elements and checklist items for global hospital antimicrobial stewardship programmes: a consensus approach. Clin Microbiol Infect.

[9] De Waele JJ, Akova M, Antonelli M, Canton R, Carlet J, De Backer D (2018). Antimicrobial resistance and antibiotic stewardship programs in the ICU: insistence and persistence in the fight against resistance. A position statement from ESICM/ESCMID/WAAAR round table on multi-drug resistance. Intensive Care Med.

[10] Kollef MH, Bassetti M, Francois B, Burnham J, Dimopoulos G, Garnacho-Montero J (2017). The intensive care medicine research agenda on multidrug-resistant bacteria, antibiotics, and stewardship. Intensive Care Med.

[11] Dyar OJ, Nathwani D, Monnet DL, Gyssens IC, Stålsby Lundborg C, Pulcini C, ESGAP Student-PREPARE Working Group (2018). Do medical students feel prepared to prescribe antibiotics responsibly: Results from a cross-sectional survey in 29 European countries. J Antimicrob Chemother.

[12] Klein Klouwenberg PM, Cremer OL, van Vught LA, Ong DS, Frencken JF, Schultz MJ (2015). Likelihood of infection in patients with presumed sepsis at the time of intensive care unit admission: a cohort study. Crit Care.

[13] Paiva JA, Mergulhão P, Gomes A, Taccone FS, Van den Abeele AM, Bulpa P (2017). Drivers and impact of antifungal therapy in critically ill patients with Aspergillus-positive respiratory tract cultures. Int J Antimicrob Agents.

[14] Yu VL, Singh N (2004). Excessive antimicrobial usage causes measurable harm to patients with suspected ventilator-associated pneumonia. Intensive Care Med.

[15] Denny KJ, de Wale J, Laupland KB, Harris PN, Lipman J (2020). When not to start antibiotics: avoiding antibiotic overuse in the intensive care unit. Clin Microbiol Infect.

[16] Rawson TM, Moore LS, Tivey AM, Tsao A, Gilchrist M, Charani E (2017). Behaviour change interventions to influence antimicrobial prescribing: a cross-sectional analysis of reports from UK state-of-the-art scientific conferences. Antimicrob Resist Infect Control.

[17] Charani E, Edwards R, Sevdalis N, Alexandrou B, Sibley E, Mullett D (2011). Behavior change strategies to influence antimicrobial prescribing in acute care: a systematic review. Clin Infect Dis.

[18] Timsit JF, Bassetti M, Cremer O, Daikos G, de Waele J, Kallil A (2019). Rationalizing antimicrobial therapy in the ICU: a narrative review. Intensive Care Med.

[19] Halpern SD (2018). Using default options and other nudges to improve critical care. Crit Care Med.

[20] Zampieri FG, Soares M, Borges LP, Salluh JI, Ranzani OT (2017). The Epimed Monitor ICU Database®: a cloud-based national registry for adult intensive care unit patients in Brazil. Rev Bras Ter Intensiva.

[21] Charani E, Castro-Sanchez E, Sevdalis N, Kyratsis Y, Drumright L, Shah N (2013). Understanding the determinants of antimicrobial prescribing within hospitals: the role of "prescribing etiquette". Clin Infect Dis.

[22] De Waele JJ, Schouten J, Dimopoulos G (2016). Understanding antibiotic stewardship for the critically ill. Intensive Care Med.

[23] Gabbay J, Le May A (2004). Evidence based guidelines or collectively constructed "mindlines?" Ethnographic study of knowledge management in primary care. BMJ.

[24] Pakyz AL, Moczygemba LR, VanderWielen LM, Edmond MB, Stevens MP, Kuzel AJ (2014). Facilitators and barriers to implementing antimicrobial stewardship strategies: Results from a qualitative study. Am J Infect Control.

[25] Moehring RW, Anderson DJ, Cochran RL, Hicks LA, Srinivasan A, Dodds Ashley ES, Structured Taskforce of Experts Working at Reliable Standards for Stewardship Panel (2017). Expert Consensus on Metrics to Assess the Impact of Patient-Level Antimicrobial Stewardship Interventions in Acute-Care Settings. Clin Infect Dis.

[26] De Bus L, Gadeyne B, Steen J, Boelens J, Claeys G, Benoit D (2018). A complete and multifaceted overview of antibiotic use and infection diagnosis in the intensive care unit: results from a prospective four-year registration. Crit Care.

[27] Saam M, Huttner B, Harbarth S, World Health Organization Expert Committee on the Selection and Use of Essential Medicines Policy, Access and Use Evaluation of antibiotic awareness campaigns.

[28] Filippini M, Ortiz LG, Masiero G (2013). Assessing the impact of national antibiotic campaigns in Europe. Eur J Health Econ.

[29] Huttner B, Goossens H, Verheij T, Harbarth S, CHAMP consortium (2010). Characteristics and outcomes of public campaigns aimed at improving the use of antibiotics in outpatients in high-income countries. Lancet Infect Dis.

[30] Australian Commission on Safety and Quality in Health Care (2018). Antimicrobial Stewardship in Australian Health Care 2018.

